# Insights into the prokaryotic communities of the abyssal-hadal benthic-boundary layer of the Kuril Kamchatka Trench

**DOI:** 10.1186/s40793-023-00522-9

**Published:** 2023-08-02

**Authors:** Susanna Gorrasi, Andrea Franzetti, Angelika Brandt, Ulrike Minzlaff, Marcella Pasqualetti, Massimiliano Fenice

**Affiliations:** 1 Laboratory of Microbiology, Department of Ecological and Biological Sciences, University of Tuscia, Viterbo, 01100 Italy; 2 Laboratory of Microbiology, Department of Earth and Environmental Sciences, University of Milano-Bicocca, Milano, 20126 Italy; 3 Senckenberg Research Institute and Natural History Museum, 60325 Frankfurt am Main, Germany; 4 Institute of Ecology, Diversity and Evolution, Goethe University, 60438 Frankfurt am Main, Germany; 5 Laboratory of Ecology of Marine Fungi - CoNISMa, Department of Ecological and Biological Sciences, University of Tuscia, Viterbo, 01100 Italy; 6 Laboratory of Applied Marine Microbiology - CoNISMa, Department of Ecological and Biological Sciences, University of Tuscia, Viterbo, 01100 Italy

**Keywords:** Bacterial communities, Archaeal communities, Deep Sea, Abyssal-hadal zone, Kuril Kamchatka Trench, 16S rRNA gene amplicon sequencing

## Abstract

**Background:**

The Kuril–Kamchatka Trench (maximum depth 9604 m), located in the NW Pacific Ocean, is among the top seven deepest hadal trenches. The work aimed to investigate the unexplored abyssal-hadal prokaryotic communities of this fascinating, but underrated environment.

**Results:**

As for the bacterial communities, we found that *Proteobacteria* (56.1–74.5%), *Bacteroidetes* (6.5–19.1%), and *Actinobacteria* (0.9–16.1%) were the most represented bacterial phyla over all samples. *Thaumarchaeota* (52.9–91.1%) was the most abundant phylum in the archaeal communities. The archaeal diversity was highly represented by the ammonia-oxidizing *Nitrosopumilus*, and the potential hydrocarbon-degrading bacteria *Acinetobacter*, *Zhongshania*, and *Colwellia* were the main bacterial genera. The α-diversity analysis evidenced that both prokaryotic communities were characterized by low evenness, as indicated by the high Gini index values (> 0.9). The β-diversity analysis (Redundancy Analysis) indicated that, as expected, the depth significantly affected the structure of the prokaryotic communities. The co-occurrence network revealed seven prokaryotic groups that covaried across the abyssal-hadal zone of the Kuril–Kamchatka Trench. Among them, the main group included the most abundant archaeal and bacterial OTUs (*Nitrosopumilus* OTU A2 and OTU A1; *Acinetobacter* OTU B1), which were ubiquitous across the trench.

**Conclusions:**

This manuscript represents the first attempt to characterize the prokaryotic communities of the KKT abyssal-hadal zone. Our results reveal that the most abundant prokaryotes harbored by the abyssal-hadal zone of Kuril–Kamchatka Trench were chemolithotrophic archaea and heterotrophic bacteria, which did not show a distinctive pattern distribution according to depth. In particular, *Acinetobacter*, *Zhongshania*, and *Colwellia* (potential hydrocarbon degraders) were the main bacterial genera, and *Nitrosopumilus* (ammonia oxidizer) was the dominant representative of the archaeal diversity.

**Supplementary Information:**

The online version contains supplementary material available at 10.1186/s40793-023-00522-9.

## Background

The oceans represent the greatest ecosystem of our planet providing a plethora of different habitats, hosting a huge diversity of organisms adapted to different environmental conditions [1]. Based on depth, the vertical profile of the oceans is divided into five zones: epipelagic (0–200 m), mesopelagic (200–1000 m), bathypelagic (1000–4000 m), abyssopelagic (4000–6000 m) and hadopelagic (> 6000 m) [1, 2]. These zones show quite diverse physicochemical features (such as salinity, temperature, light, pressure and nutrient concentration) that create distinct conditions affecting microbial communities [3]. Therefore, the characterization of microbial communities across the oceanic zones is important to understand their vertical distribution and ecological role in marine processes, including biogeochemical cycles.

The hadal zones, the deepest part of the oceans composed almost exclusively of trenches, are among the least studied habitats on the planet for their microbial assemblages. Various studies have regarded the isolation of piezophilic and hyperpiezophilic microorganisms from trenches worldwide [4–8]. These include species of archaea, bacteria and fungi including some recently described novel taxa: *Muricauda abyssi, Paraoceanicella profunda, Abyssibius alkaniclasticus* [9–11] As for the profiling of prokaryotic communities, the investigation has been principally focused on the Mariana Trench [3, 12–26] and to a lesser extent on other trenches (e.g., Puerto Rico, Japan, Kermadec, Izu-Ogasawara, Atacama, New Britain and Yap trenches) [16, 17, 27–34]. If compared to the literature available for the trench fauna, works that have reported the prokaryote intra-trench variation along the water column are scarce [14, 15, 17, 20–22]. Still fewer are the works addressing the inter-trench variability of the prokaryotic diversity; however, most of the studies analyzed the vertical/spatial intra-trench variation of prokaryotes in the Mariana Trench [e.g., 16,17,19,23,24,33]. Meanwhile, the prokaryotic variability within the other trenches or inter-trenches remains poorly investigated. Differences in prokaryotic diversity may exist at different depths within the same trench, but also among different trenches, which are geographically separated habitats with distinctive environmental conditions [4, 29, 35]. Thus, more studies investigating the intra- and inter-trench variation of the prokaryotic communities are needed to extend the knowledge of the community variability of these unique extreme habitats.

The Kuril–Kamchatka Trench (KKT) or Kuril Trench is among the top seven deepest (> 9000 m) hadal trenches [36, 37]. It is a linear (slightly convex) depression of ca. 2089 km in length (surface area of 130,985 km^2^ and maximum depth of 9604 m) located in the northwest Pacific Ocean, which joins the Japan Trench (creating a hadal continuum) in the south-west and meets the Aleutian Trench in the north-east [36, 38]. The KKT region is affected by various oceanic circulations having different and sometimes opposite directions, resulting also in interactions between cold and warm waters; the trench upper waters are mainly affected by the Oyashio current, whereas two bottom currents affect the deep waters [39, 40]. Actually, water circulation involves only the layers above the hadal region of the trench, whereas in the deeper V-shaped depression no current has been recorded; the Lower Circumpolar Deep Water is the deepest current (~ 4000 m depth) reaching the KKT region [41, 42]. The trench, together with the interconnected Kuril Basin is placed in one of the most productive oceanic areas in the world [43].

Although the KKT region has been investigated since 1949 (by the soviet *Vitjaz* expeditions; [44]), the studies were principally focused on characterizing its oceanographic, chemical and physical features and on investigating the productivity, plankton and benthic fauna distribution [45–47]. Studies carried out on the KKT microbial diversity are definitely scant. One work has been focused on bacteria from hadal bivalve gills [48], and, to the best of our knowledge, only two works (involving sampling in the KKT region or neighboring areas) have investigated the prokaryotic diversity of the site [49, 50]. However, these studies limited the investigation to the upper layers (from the surface to the bathyal zone) considering a maximum depth of 3000 m. Other studies evidenced that many macrofaunal species of the trench and nearby areas floors host chemosynthetic endosymbiotic, indicating that the bottom communities are sustained by the bacterial chemosynthetic organic carbon production [43, 51]: this seems to be a common feature in bottom oceanic fauna [52].

The Aim of this work was an in-depth investigation of the unexplored prokaryotic communities of the abyssal-hadal zone (depth range 5146–9540 m) of the Kuril-Kamchatka Trench. The study was carried out on the benthic-boundary layer waters sampled in various sites of the trench and of the adjacent abyssal plan.

## Materials and methods

### Sample collection and characterization

**Fig. 1:**
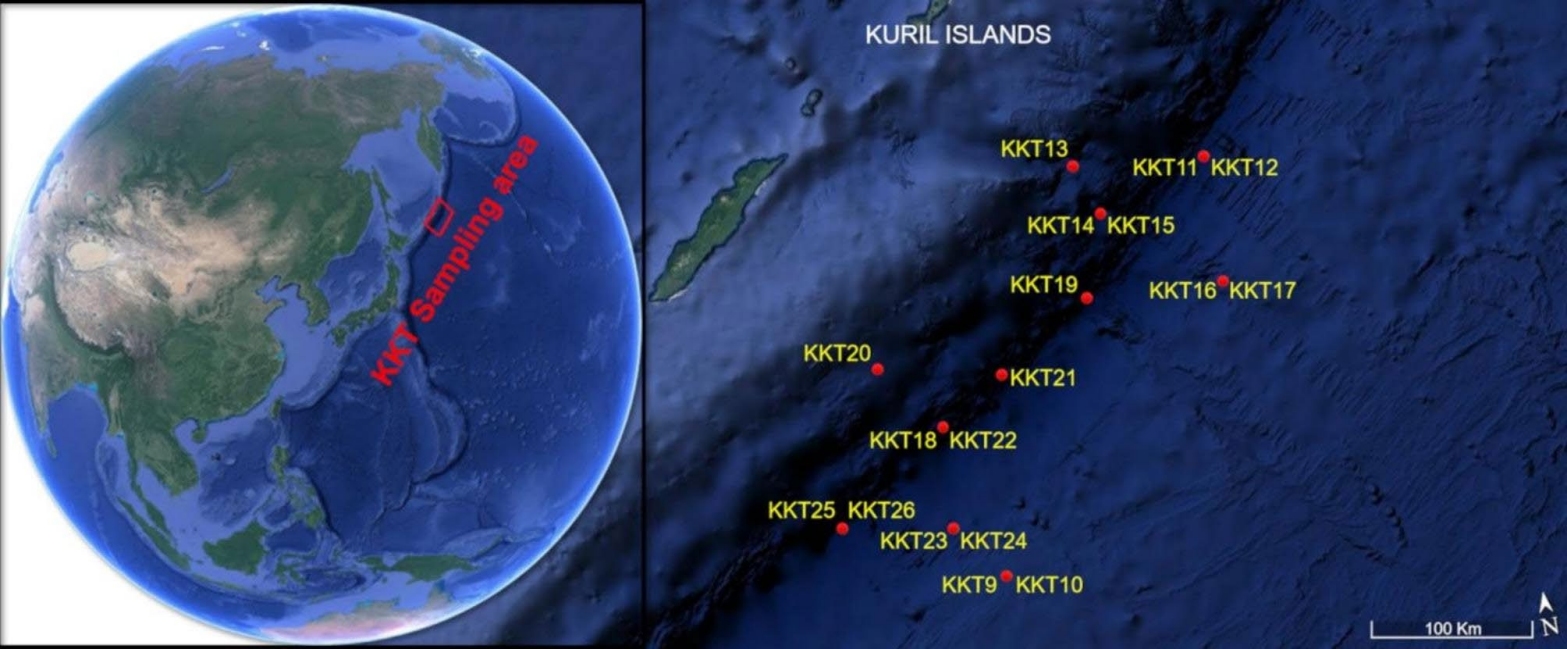
Kuril-Kamchatka Trench map with indication of the sites (red points) where samples were collected. The map was generated using Google Earth Pro version 7.3.1. and graphically edited

The eighteen water samples used for the survey were collected in various abyssal and hadal areas within the Kuril-Kamchatka Trench region (Northwest Pacific Ocean) between August 16 and September 26, 2016, aboard the deep-ocean Research Vessel (RV) *Sonne*, during the German-Russian expedition KuramBio II (Kurile-Kamchatka Biodiversity Studies II) (Fig. 1).

The sampling involved different sites within the trench and in the nearest zones of the adjacent abyssal plain (depth range 5146–9540 m) (Table 1). The samples were representative of the benthic boundary layer (water layers that are directly adjacent to the ocean bottom and can contain particles derived from the resuspension of surface sediments; [15]). For each depth, one sample was taken from the water overlaying (~ 30 cm) one of the cores collected by the OKTOPUS® multicorer (GmbH, Kiel, Germany) equipped with 12 transparent acryl-glass-cylinders (length 60 cm, inner diameter 9.5 cm).

**Table 1:** Sampling site coordinates and related depths

Sample	Coordinates	Depth
	(Latitude; Longitude)	(m)
KKT9	43° 49.19’ N; 151° 45.59’ E	5146.6
KKT10	43° 49.19’ N; 151° 45.59’ E	5146.7
KKT11	45° 50.87’ N; 153° 47.99’ E	8254.7
KKT12	45° 50.87’ N; 153° 47.99’ E	8255.2
KKT13	45° 55.23’ N; 152° 47.46’ E	6065.0
KKT14	45° 38.60’ N; 152° 55.91’ E	7136.7
KKT15	45° 38.60’ N; 152° 55.92’ E	7134.6
KKT16	45° 09.99’ N; 153° 45.41’ E	5741.2
KKT17	45° 10.00’ N; 153° 45.43’ E	5739.4
KKT18	44° 39.89’ N; 151° 28.11’ E	8221.1
KKT19	45° 12.94’ N; 152° 42.83’ E	9498.9
KKT20	45° 01.36’ N; 151° 02.89’ E	5211.3
KKT21	45° 01.36’ N; 151° 02.89’ E	5217.4
KKT22	44° 39.90’ N; 151° 28.10’ E	8224.0
KKT23	44° 06.85’ N; 151° 25.54’ E	6517.5
KKT24	44° 06.84’ N; 151° 25.55’ E	6515.8
KKT25	44° 12.40’ N; 150° 36.02’ E	9538.6
KKT26	44° 12.39’ N; 150° 36.01’ E	9540.2

For each sample, 1 L of seawater was filtered on sterile membranes, which were maintained frozen until DNA extraction (that was carried out on land).

### DNA extraction, amplicon libraries and sequencing

Total DNA was extracted from filters using the ZR Fungal/Bacterial DNA MiniPrep kit (Zymo Research Corp., Irvine, CA, USA) according to the manufacturer’s instructions.

The multiplexed amplicon libraries for *Bacteria* and *Archaea* (which were prepared and sequenced separately) were obtained using a dual PCR amplification protocol.

The first PCR was performed using primers modified by adding a 6-bp barcodes to allow the parallel processing of multiple samples, as previously reported [53, 54]. For both amplicon libraries, the first PCR was carried out in 3 × 80 µL volume reactions using the Phusion® High-Fidelity DNA Polymerase (NEB Inc., Ipswich, MA, USA) and 1 µM of each primer. For the bacterial community analysis, the V5–V6 hypervariable regions of the 16 S rRNA gene were targeted using the primer set 783 F/1046R [55, 56]. The PCR cycling conditions were: 94 °C for 4 min; 28 cycles at 94 °C for 50 s, 47 °C for 30 s, and 72 °C for 30 s; and a final extension at 72 °C for 5 min. For the archaeal community analysis, the V3 hypervariable region of the 16 S rRNA gene was targeted using the primer set Arch349F/571R [57, 58]. The PCR cycling conditions were: 98 °C for 30 s; 10 cycles at 98 °C for 10 s, 68 °C for 20 s, and 72 °C for 15 s; 35 cycles at 98 °C for 10 s, 58 °C for 15 s, and 72 °C for 15 s; and a final extension at 72 °C for 2 min. The amplicons were purified by Wizard SV Gel and PCR Clean-up System (Promega Corporation, Madison, WI, USA) and quantified using Qubit 2.0. After the purification, nine amplicon samples (identifiable by a unique barcode pair combination) were pooled to obtain a single library.

The second step of the library preparation, consisting of a second PCR for the addition of the standard Nextera indexes (Illumina, Inc., San Diego, CA, USA), the DNA quality evaluation and quantification, and the subsequent amplicon library sequencing were carried out at Nuova Genetica Italiana SRL (Monza-Brianza, Italy). The amplicon libraries were sequenced by Illumina MiSeq (Illumina Inc., San Diego, CA, USA) using a 2 × 250 bp paired-end protocol.

### Sequence processing and data analysis

Sequence processing and data analysis were carried out as previously reported [59, 60]. The raw reads obtained from the sequencing were demultiplexed according to the indices and internal barcodes, and processed using the UPARSE pipeline [61]. Forward and reverse reads were merged with perfect overlapping and quality filtered using the default parameters. Suspected chimeras and singletons (unique sequences on the whole data set) were removed. Operational Taxonomic Units (OTUs) were defined on the whole data set clustering the sequences at 97% of similarity, and a representative sequence was defined for each cluster. The OTU abundances per sample were estimated by mapping the sequences to the OTU representative sequences (at 97% of similarity). The taxonomic analysis was performed using RDP classifier [62], applying the 50% confidence cut-off as suggested for short sequences [63].

The sequence data have been submitted to the GenBank database under the accession numbers PRJNA799316 and PRJNA799327.

### Statistical methods

Sequence processing and data analysis were carried out as previously reported [64]. The coverage of each sample was estimated by the Good’s method [65]. For both *Bacteria* and *Archaea* libraries, rarefaction curves were calculated and plotted to visualize OTU richness as a function of increasing sequencing depth. The Shannon [66] and Gini inequality [67] indices (calculated on rarefied data) were used to describe the α-diversity. The analyses of α-diversity indices were performed using linear models after checking by visual inspection of residual plots (details not shown) that no relevant deviation from model assumption had occurred.

β-diversity analyses were performed on the non-rarefied samples [68] using for each OTU the number of the relating sequences retrieved in each sample as estimation of its abundance in the sample. OTU abundances per sample were transformed using the Hellinger method [69]. The Redundancy Analysis (RDA) was performed to investigate the variation of community structure in relation to the depth (fundamental parameter for the trench environments); the analysis significance was assessed through 9999 permutations. For both datasets (*Bacteria* and *Archaea*), the clustering analysis (complete linkage) was performed based on Hellinger distance data. The abundance variation of the most abundant genera (having at least 20,000 (bacterial taxa) and 9,000 (archaeal taxa) sequences in the whole dataset) in relation to the depth was investigated by Generalized Linear Models (GLMs).

The GLMs were performed assuming a Poisson distribution and corrected for overdispersion. We accounted for multiple statistical tests by correcting GLM significance (P-value) according to the False Discovery Rate (FDR) procedure [70]. All the statistical analyses were performed in R environment (R 3.4.2, R Core Team, 2018) using VEGAN, BIODIVERSITYR, MULTTEST, and MULTCOMP packages.

The Functional Annotation of Prokaryotic Taxa software program (FAPROTAX, Version 1.2.6) [71] was used to predict the potential functions of the KKT abyssal-hadal communities, based on the functional annotation information of the cultured prokaryotes.

The network analysis was used to investigate the co-occurrence patterns among bacterial and archaeal OTUs which mainly contribute to the community structure. To reduce the networking complexity, the analysis was carried out only on OTUs with relative abundance (Ra) ≥ 1% at least in one sample. The Spearman correlation matrix (Spearman’s correlation coefficient (ρ) ≥ |0.8|; q values < 0.01 after Bonferroni correction) was calculated on the compositional dataset using the statistical software Systat 8.0 (Systat Software Inc., Point Richmond, CA, USA). The Visone 2.21 free software (https://visone.ethz.ch/index.html) was used for the network exploration and visualization [72].

## Results and discussions

This work was carried out within the German-Russian expedition KuramBio II which was specifically planned to increase the knowledge about this oceanic ecosystem, started with a previous oceanic campaign (Kurambio). The main aim of both KURAMBIO expeditions was to study the animal species composition and distribution at the trench bottom, and to evaluate the faunal relationships in the studied deep-sea ecosystems [43]. Samples from KKT, which is practically unknown under the microbiological point of view, were selected for this study due to extreme environmental conditions of the trench and, in particular, its high hydrostatic pressure. During the oceanographic expedition, the environmental parameters related to the benthic-boundary layer samples were not registered due to both technical and logistical reasons.

### Bacterial community profiling

After suspected chimera (n = 12,762) and singleton (n = 635) removal, a total of 1,205,906 bacterial mapping sequences were obtained; the Good’s index values (97.9–99.7) indicated a good coverage for the libraries. Overall, 4628 OTUs were defined through the clustering sequence process.

The bacterial OTUs were assigned to 27 phyla, 58 classes, 108 orders, 234 families and 453 genera. Unassigned OTUs ranges were 2.4–9.1%, 5.6–14.1%, 24.9–41.7%, 33.9–50.7%, and 44.9–68.5% at phylum, class, order, family, and genus level, respectively.

**Fig. 2:**
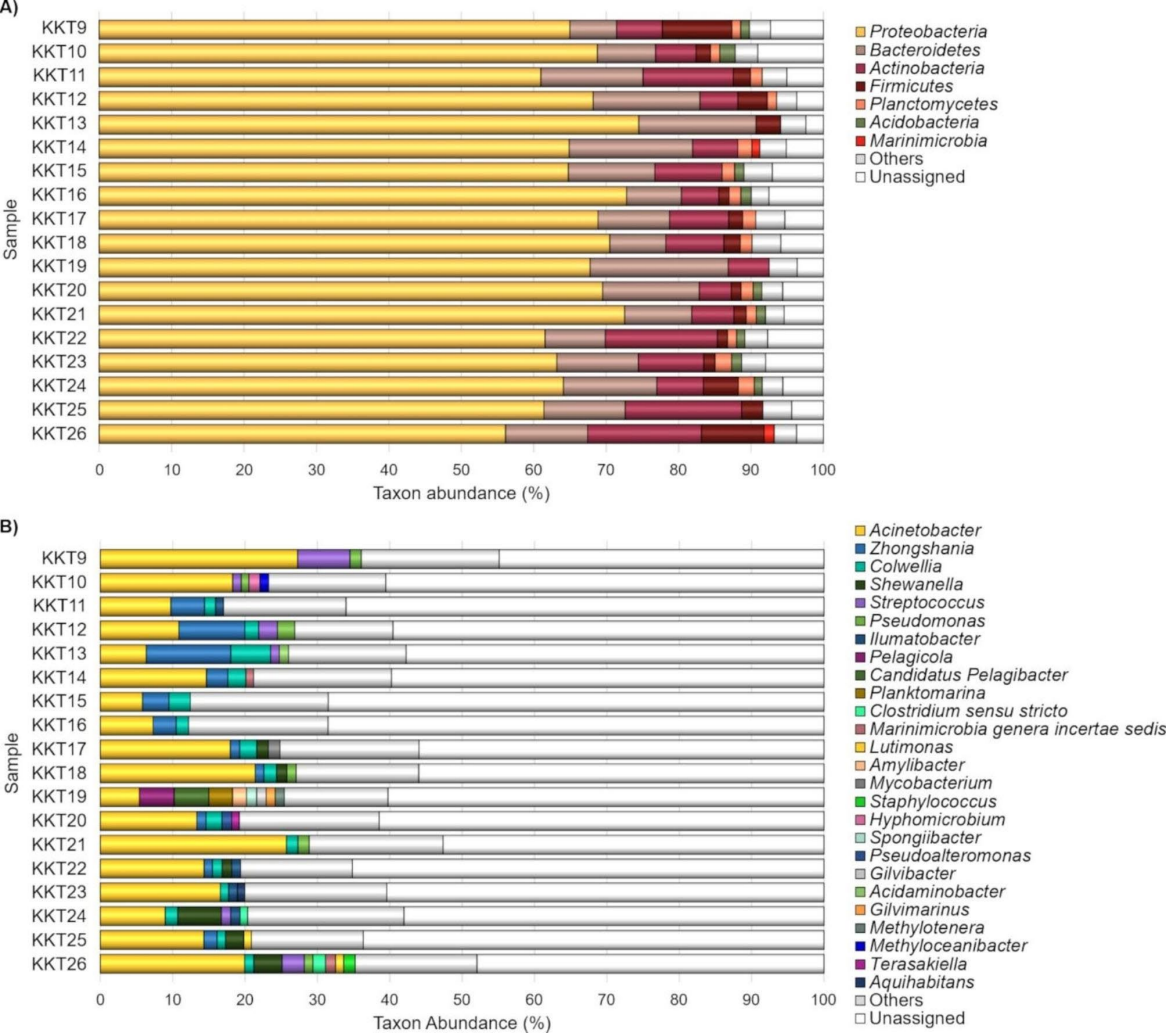
Bacterial composition of the KKT samples at the phylum **(A)** and genus **(B)** level. The bar plots show the distribution of the major taxa (Ra ≥ 1% in at least one sample); taxa with Ra < 1% were gathered in “Others”

At the phylum level, the major bacterial taxa (Ra ≥ 1% in at least one sample) were *Proteobacteria*, *Bacteroidetes*, *Actinobacteria*, *Firmicutes, Planctomycetes, Acidobacteria* and *Marinimicrobia* (Fig. 2A). *Proteobacteria*, *Bacteroidetes* and *Actinobacteria* were the most represented taxa over all the KKT samples, and except for *Actinobacteria* in KKT13 (6065 m depth), they were always recorded with Ra ≥ 1%. *Proteobacteria* (56.1–74.5%; mean abundance of 66.42%) dominated the abyssal-hadal KKT bacterial communities, followed by *Bacteroidetes* (6.5–19.1%; mean abundance of 11.69%) and *Actinobacteria* (0.9–16.1%; mean abundance of 8.11%). *Firmicutes* (0.3–9.6%) had Ra ≥ 1% in most of the samples (15 out of 18 samples); its mean abundance was 2.9% and it showed the highest abundances (≥ 8.6%) in KKT9 (~ 5146 m depth) and KKT26 (~ 9540 m depth). *Planctomycetes* (0.8–2.3%) and *Acidobacteria* (0.3–2.1%) showed mean abundance of 1.5 and 1% and they were recorded among major taxa in 78% and 50% of samples. *Marinimicrobia* was detected usually as minor taxon, except in KKT14 and KKT26, sampled at ~ 6516 and ~ 9540 m, respectively.

As expected, the KKT bacterial communities of the abyssal-hadal zone studied in our work differed in both phyla presence and abundance from those of the shallower layers reported by Li et al. [50]. These authors carried out a sampling campaign with stations located around the Sea of Okhotsk and North Western Subarctic Pacific Ocean, including the KKT area affected by the East Kamchatka Current. Samples were mainly taken from the surface and two of them were collected from the mesopelagic (1000 m) and bathypelagic (3000 m) waters. Meso- and bathy-pelagic waters differ from those of the abyssal and hadal ones; they are more unstable and influenced by the currents.

The sample taken at 1000 m, that is generally considered the beginning of piezosphere, should represent a depth where piezophilic microorganisms are considered to acquire advantages over the piezosensitive. Here the waters, should be characterized mainly by the presence of piezotolerant microorganisms than piezophiles, which have their optimal growth conditions at a depth of around 4000–6000 m. The sample taken at 3000 m should not contain piezosensitive micoorganisms but only piezotolerant and piezophilic ones. In any case, in both samples, no extreme-piezophiles should be present [73, 74]. Moreover, a certain taxon could be found at different depths but it could include different species or even strains showing different adaptations to the hydrostatic pressure. For the mentioned reasons the comparison between the present work and that of Li et al. [50] could be considered useful for an intra-trench prokaryotic biodiversity evaluation. Also these authors observed a preponderance of *Proteobacteria*, which seems to be the main bacterial phylum at almost all depths being its Ra always higher than 40%. In their surface waters, there was also a high presence of *Bacteroidetes* and *Cyanobacteria* and sometimes of *Verrucomicrobia*. In the deeper waters, the biodiversity at the phylum level was higher than that of the surface waters and characterized by the presence also of *Planctomycetes, Marinimicrobia, Actinobacteria*, and *Acidobacteria.*

Since our water samples are representative of the benthic boundary layer (presenting also microorganisms from the sediment surface resuspension; [15]) a useful comparison could be done with the prokaryotic communities of both waters and sediments of other trenches (e.g., Mariana Trench, Japan Trench, Puerto Rico Trench, Yap Trench).

Various studies agreed with the general predominance of *Proteobacteria* in the abyssal-hadal zones [e.g.,14,16,18,22,27,29,31], whereas others reported dominance of different taxa. For example, in the work of Nunoura et al. [12], carried out in the Challenger Deep in the Mariana Trench, *Proteobacteria* dominated in the bathyal zone (1000–2000 m) and in the hadal zone at 10,000 m, while *Bacteroidetes* dominated in the abyssal-hadal zone from 6000 to 9000 m. In the same area, Nunoura et al. [13] observed a very strong abundance of *Protobacteria* in hadal waters at 10,257 m, whereas *Chloroflexi* and into a second extent *Bacteroidetes*, *Planctomycetes*, *Marinimicrobia* and *Gemmatimonadetes* were the most abundant taxa in the sediments. In a later work [21], characterizing the Challenger Deep prokaryotic communities, in most of the samples taken from the hadal waters, *Marinimicrobia* was the most abundant bacterial phylum. Dominance of *Marinimicrobia* was found also in hadal waters and sediments from other sites [12, 13, 20, 30, 75]. In our case, except for KKT14 (~ 7137 m) and KKT26 (~ 9540 m), this phylum was always a rare taxon. Overall, an inter-trench diversification of the bacterial community assemblages is evident already at the phylum level.

However, a possible comparison of data related to different trenches obtained in different periods (years and/or months), would be more meaningful if referred to samples taken in stable abyssal-hadal zones rather than with those obtained from the upper less stable layers subject to various perturbations such as those caused by oceanic currents.

At the genus level, a very high abundance of unassigned OTUs (from 44.9 to 68.5%) occurred **(**Fig. 2B). As underlined by others [76, 77], a high proportion of unassigned microbial sequences in deep seas suggests that the majority of the microbial diversity remains largely unknown. However, the sequence length does not allow to confidently state whether the unassigned sequences could be attributed to known genera or to putative new taxa. To overcome this problem the complete 16S rRNA gene could be sequenced in a subsequent study.

Even if the subsequent observations are based on relative abundance data calculated from the sole OTUs assigned at genus level, it is still possible to make some general considerations.

The bacterial OTUs were assigned to 453 genera; among them, 26 taxa showed Ra ≥ 1% in at least one sample. The most represented genus was *Acinetobacter*, followed by *Zhongshania* and *Colwellia*.

*Acinetobacter* was the sole genus representing a major taxon in all samples, with no significant abundance variation in relation to the depth, as suggested by the GLM analysis (P_FDR_ > 0.05). It was recorded with abundances spanning from 5.4% (in KKT19; ~9499 m depth) to 27.3% (in KKT9; ~5147 m depth). Moreover, its highest abundances (≥ 20%) were recorded both in the upper layers of the abyssal zone (~ 5147 and ~ 5217 m depth) and in the deep layers of the hadal zone (~ 8221 and ~ 9540 m depth). *Acinetobacter* has been found in a wide array of environments and includes various species having different ecological roles, from pathogeny to biodegradation of xenobiotics [78]. It has been revealed in marine sediments and waters, from the surface to the deepest zones, including some oceanic trenches [31, 79–88]. The metabolic features and the potential ecological functions of some deep-sea members of *Acinetobacter* indicated that they can be involved in sulphur and metal oxidation, and hydrocarbon degradation [80, 84, 88–90].

*Zhongshania* and *Colwellia* were detected in all samples, but they showed Ra < 1% in six (KKT9, KKT10, KKT19 KKT21, KKT23, and KKT26) and two (KKT9 and KKT19) samples, respectively. Also for these genera, a significant abundance variation according to the depth was not evidenced (P_FDR_ > 0.05). *Zongshania* ranged from 0.1 to 11.7%, and it showed the highest abundances (> 9%) at ~ 6065 m and ~ 8255 m. *Colwellia* showed abundances in the range 0.4–5.5% with the highest abundance at ~ 6065 m. *Zhongshania*, usually detected as major taxon in the KKT abyssal and hadal samples, has various psychrophilic and psychrotolerant members isolated from diverse marine environments (from Antarctic coastal waters to tidal sediments of the Yellow Sea), but only up to 500 m depth [91–94]. No information concerning its presence in the deepest oceanic zones is available and there is scarce knowledge about its behavior in relation to increasing hydrostatic pressure. Only one work tested *Zhongshania* strains (isolated from coastal seawater samples) at increasing hydrostatic pressures, evidencing their piezotolerant phenotype [95]. Hence, as far as we know, our work is the first reporting the presence of these bacteria in the deepest oceanic zones (5146–9540 m depth) possibly confirming the existence of piezophilic/piezotolerant members. It is interesting to note that various members of the genus *Zhongshania* are hydrocarbon-degrading bacteria [93, 95–97]. In addition, a sequential cooperation among oil-degrading strains of *Zhongshania* and *Colwellia* was reported: *Zhongshania* plays a fundamental role in the initial n-alkanes biotransformation that is completed by *Colwellia*, through different metabolic pathways including also the beta‑oxidation [96].

**Fig. 3:**
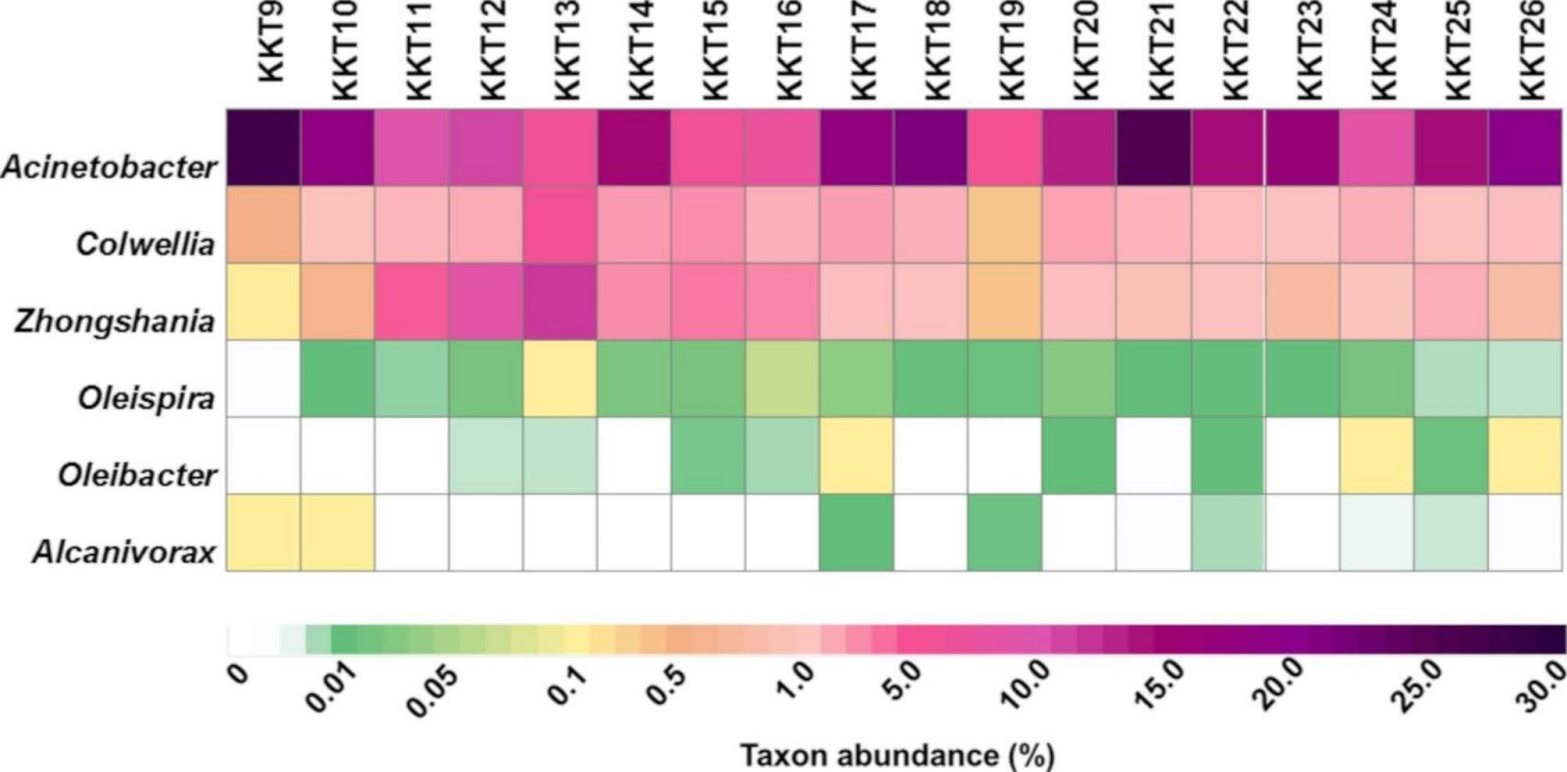
Occurrence of *Acinetobacter*, *Zhongshania*, *Colwellia* and genera of obligate hydrocarbonoclastic bacteria (*Oleispira*, *Oleibacter* and *Alcanivorax*) across the KKT abyssal and hadal samples

Therefore, it is possible to speculate that the most represented bacteria found in the KKT abyssal-hadal zone are potentially hydrocarbon-degraders. In addition, the presence of well-known obligate hydrocarbonoclastic bacteria (A*lcanivorax, Oleibacter* and *Oleispira*; [98]) in some KKT samples, although as minor taxa (Fig. 3), might reinforce this hypothesis, indicating also that hydrocarbons could represent a significant carbon source in these deep environments.

Presence of hydrocarbons at the trench bottom could be due both to xenobiotic (e.g., oil spill falls) and biotic (from heterotrophic and autotrophic organisms) sources revealed in sinking particles [18]. Thus, the occurrence of the hydrocarbonoclastic bacteria in the KKT samples is consistent with what found for other trench environments: the deepest zones support the growth of these heterotrophic microorganisms that locally contribute to hydrocarbon recycle at the bottom of the ocean [3, 99].

A list of the bacterial genera found in the abyssal-hadal zones of KKT and other main trenches was supplied to provide a more detailed comparison (Additional file 2: Table S1).

### Archaeal community profiling

After suspected chimera (n = 3613) and singleton (n = 977) removal, a total of 1,226,044 archaeal mapping sequences were obtained; the Good’s index values (99.4–99.8) indicated a good coverage for the libraries. Overall, 3362 OTUs were defined through the clustering sequence process.

**Fig. 4:**
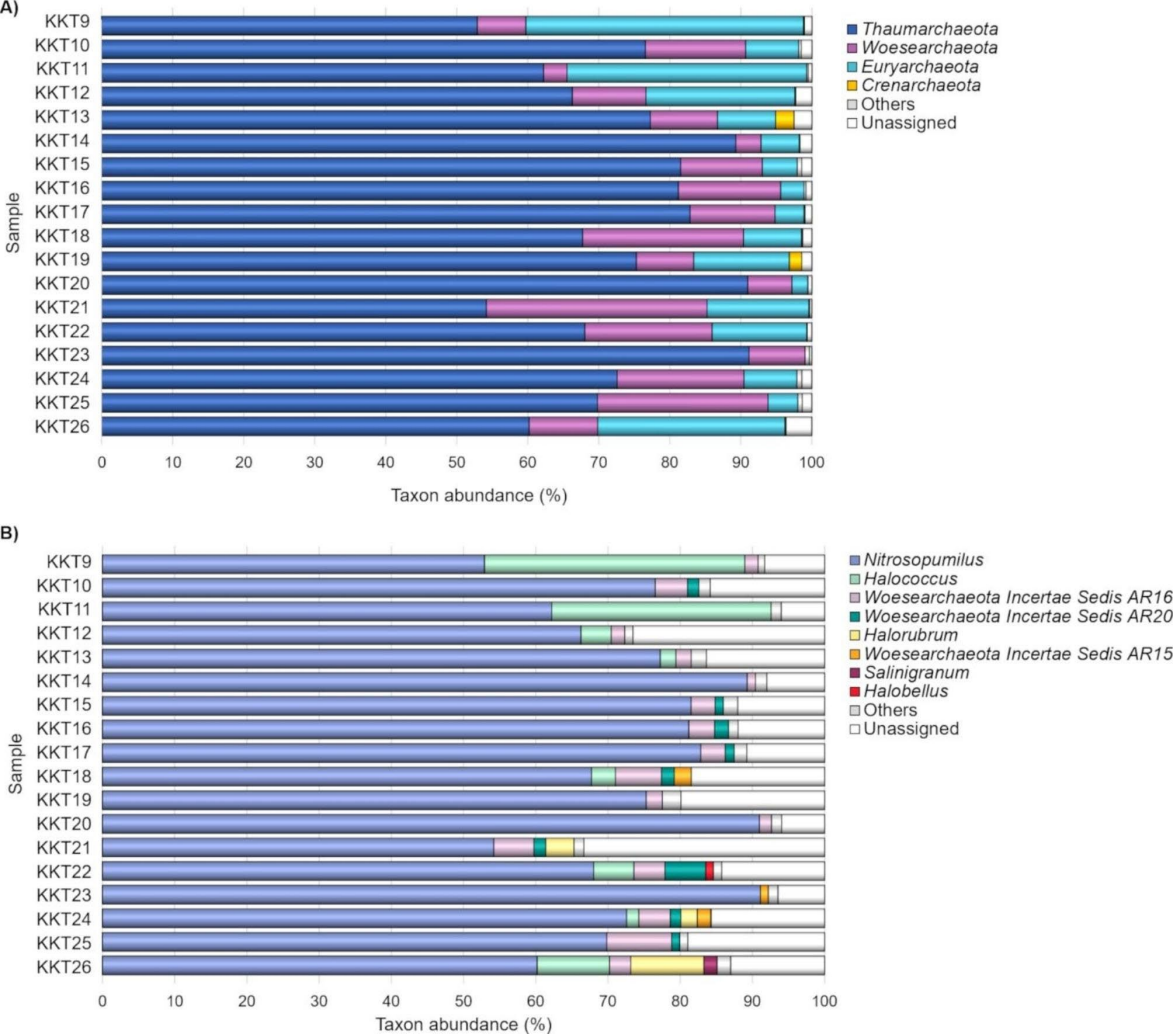
Archaeal composition of the KKT samples at the phylum **(A)** and genus **(B)** level. The bar plots show the distribution of the major taxa (Ra ≥ 1% in at least one sample); taxa with Ra < 1% were gathered in “Others”

Since for some archaeal genera the lineage is currently not fully defined, we report only the summary of OTU assignation obtained at phylum and genus levels. The archaeal OTUs were assigned to 5 phyla and 21 genera. Unassigned OTUs ranged from 0.3 to 3.6% and from 6 to 33.3% at phylum and genus level, respectively. The archaeal phyla detected were *Thaumarchaeota*, *Woesearchaeota*, *Euryarchaeota*, *Crenarchaeota*, and *Diapherotrites* (Fig. 4A). Among them, *Diapherotrites* was the sole phylum representing always a minor taxon (it was present in 13 out of 18 samples with Ra in the range 0.001-0.2%). *Crenarchaeota* was detected as a major genus only in two samples (1.7 and 2.6% in KKT19 and KKT13, respectively). Members of this phylum have been detected in various oceanic environments, often representing an abundant archaeal fraction in oxygenated deep waters and surface sediments [100].

*Thaumarchaeota* (52.9–91.1%), *Woesearchaeota* (3.3–31.1%), and *Euryarchaeota* (0.6–39.1%) were the most represented taxa over all samples; except for *Euryarchaeota* in KKT23 (~ 6518 m depth), they were always detected with Ra ≥ 1%.

The archaeal community assemblages revealed for the KKT samples are consistent with those recognized by Jing et al. [15] in the benthic boundary layer of the Mariana Trench. As already observed for the bacteria, also the archaeal communities of the KKT abyssal-hadal zone differ from those of the shallower layers in phyla presence/abundance [50]. However, Li et al. [50] outcomes confirmed *Thaumarchaeota* and *Euryarchaeota* as the main archaeal phyla in the upper layers (1000 and 3000 m depths) as well.

The current study evidenced that *Thaumarchaeota* dominated the archaeal communities of the KKT abyssal-hadal zone, showing a notable preponderance (Ra ≥ 80%) in one third of the samples. Other studies revealed a dominance of this taxon in the archaeal communities of both waters and sediments across various deep oceanic habitats, where they are followed by *Woesearchaeota* (mainly in sediments) and *Euryarchaeota* (mainly in waters) [13, 14, 16, 21–24, 28, 29, 31, 32].

Marine *Thaumarchaeota* are well-known chemolithoautotrophs (spanning from mesopelagic to hadal environments) involved in dark primary production, with key roles in nitrogen and carbon biogeochemical cycles [101].

**Fig. 5:**
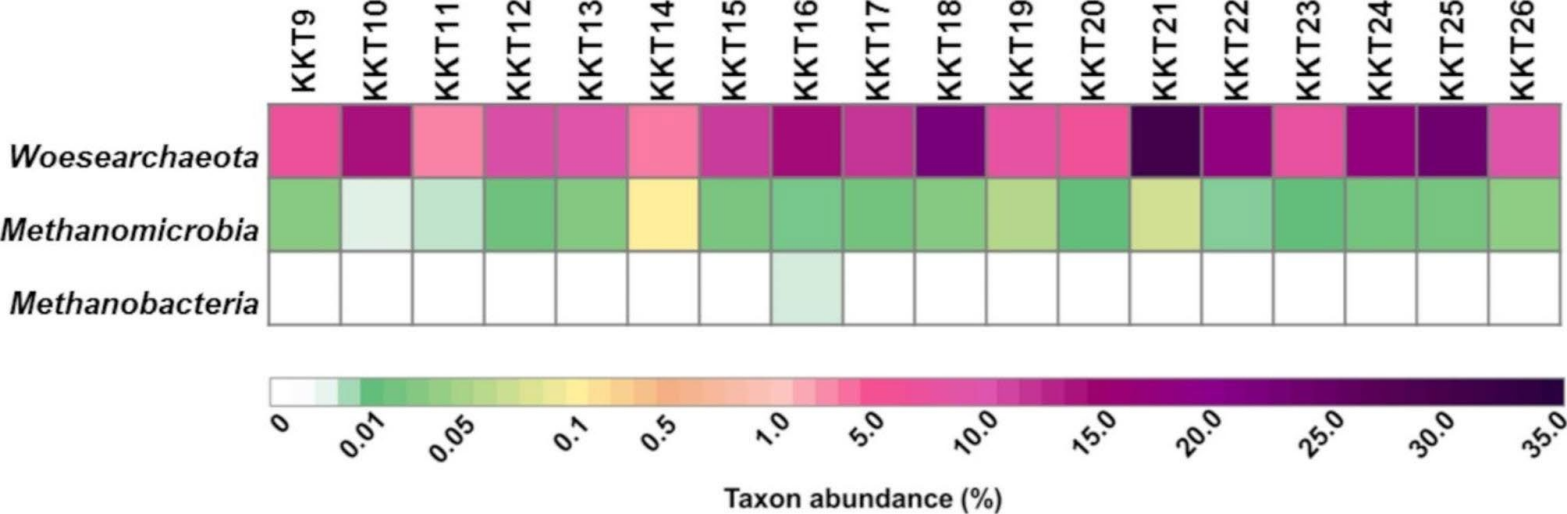
Occurrence of *Woesearchaeota* and classes of methanogens *(Methanomicrobia* and *Methanobacteria*) across the KKT abyssal and hadal samples

Marine *Euryarchaeota* has been divided in three major groups (MG-II, MG-III, and MG-IV). MG-II members represent the dominant fraction of the archaeal communities within the euphotic zone, but they have also been found in deep-sea waters. MG-III and MG-IV are considered to be rare components of deep-sea communities [102]. Little is known about the ecological partitioning and physiology of these groups; in addition, the scarce knowledge of their metabolic competencies means their impact on biogeochemical cycles still speculative. However, MGII and MGIII have been suggested to be involved in the degradation of high-molecular-weight compounds, therefore affecting primary the carbon biogeochemical cycle [103]. *Woesearchaeota* has been found in different environments, including marine waters/sediments and hydrothermal vents [104]; however, its biodiversity, distribution and metabolisms remain largely unknown [105]. Nevertheless, it has been suggested that most of *Woesearchaeota* might have a mainly anaerobic or fermentation-based lifestyle, also under potential syntrophic/mutualistic partnership with other microbial groups [104, 105]. It has been demonstrated that *Woesearchaeota* tend to co-occur in a consortium with anaerobic methanogens of *Methanomicrobia* and *Methanobacteria*, and show a possible role in the hydrogen cycle [105]. Also our survey revealed that *Woesearchaeota* and *Methanomicrobia* were concurrently detected at all depths (Fig. 5); thus, a potential syntrophic partnership between these two taxa could be reasonably suggested.

The archaeal genera detected as major taxon in at least one KKT sample were *Nitrosopumilus*, *Halococcus*, *Woesearchaeota Incertae Sedis AR16*, *Woesearchaeota Incertae Sedis AR20*, *Halorubrum*, *Woesearchaeota Incertae Sedis AR15*, *Salinigranum* and *Halobellus* (Fig. 4B).

Among them, the most represented taxa were *Nitrosopumilus* (52.9-91-1%), *Halococcus* (0.1–36%), and *Woesearchaeota Incertae Sedis AR16* (0.7-9%). All the most abundant genera did not show significant differences in abundances in relation to the depth (P_FDR_ > 0.05), indicating a general ability to cope with different hydrostatic pressure conditions. *Woesearchaeota Incertae Sedis AR16* was among the major taxa in 16 samples out of 18, it had mean abundances of 3.3% with the highest abundance (9%) in KKT25 (~ 9539 m depth). *Halococcus* was recorded with Ra ≥ 1% in eight samples; it showed high abundance (30.4 and 36%) in KKT9 and KKT11 collected at ~ 5147 m and ~ 8224 m depth, respectively.

Hence, the archaeal communities of the KKT abyssal-hadal zone were dominated by *Nitrosopumilus*, which accounted at least for 99.98% of *Thaumarchaeota* that was the most abundant archaeal phylum. Therefore, *Nitrosopumilus* represented the main fraction of the whole KKT archaeal diversity.

**Fig. 6:**
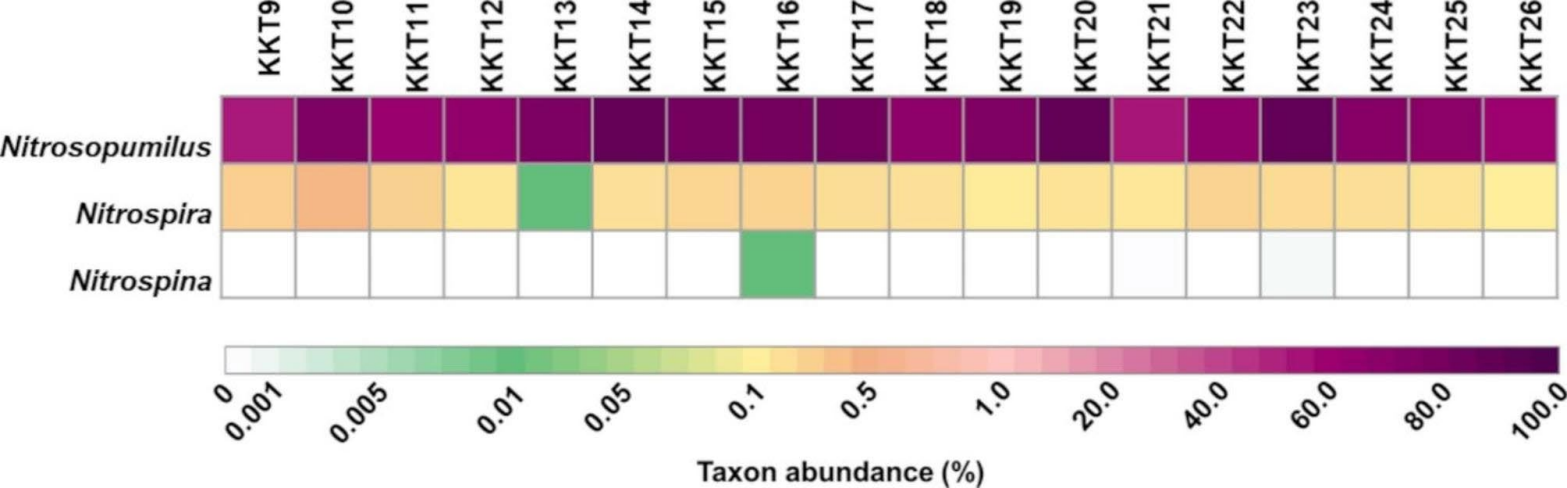
Occurrence of *Nitrosopumilus* and genera of nitrite oxidizing bacteria *(Nitrospira* and *Nitrospina*) across the KKT abyssal and hadal samples

*Nitrosopumilus* is a globally abundant marine genus with high biogeochemical significance playing a critical function in global element cycling particularly for carbon (due to their chemolithoautotrophy) and nitrogen (for their role in ammonia oxidation) [3, 14, 16, 17, 19–21, 23, 34, 106]. These chemolithoautotrophs and ammonia-oxidizing archaea are considered key primary producers principally when photoautotrophy is not possible [107, 108]. Moreover, the ammonia-oxidizing archaea are generally found in co-presence with nitrite-oxidizing bacteria in most ecosystems [109, 110]. In the oceans, ammonia is mainly oxidized by ammonia oxidizing archaea (among them *Nitrosopumilus*), nitrite is further oxidized to nitrate by nitrite oxidizing bacteria, primarily belonging to the phylum *Nitrospinae* and to a lesser extent to the genera *Nitrococcus* and *Nitrospira* [109]. The presence of nitrite oxidizing bacteria of *Nitrospina* (phylum *Nitrospinae*) and *Nitrospira* was revealed (although with Ra ≤ 1%) in the KKT samples (Fig. 6), suggesting a potential partnership with the ammonia oxidizing archaeon *Nitrosopumilus*.

A list of the archaeal genera found in the abyssal-hadal zones of KKT and other main trenches was supplied to provide a more detailed comparison (Additional file 2: Table S2).

### Alpha- and Beta-diversity of the bacterial and archaeal communities

Rarefaction curves were separately evaluated for *Bacteria* and *Archaea* datasets (Additional file 1: Fig. S1) to perform dataset rarefaction to the minimum library depth.

The α-diversity indices of the bacterial and archaeal communities of the KKT abyssal and hadal samples are reported in Table 2. Summarizing, the Number of OTUs ranged from 716 to 2163 and from 243 to 1031 for the bacterial and archaeal libraries, respectively. The Shannon diversity index ranged from 2.686 to 5.818 and from 3.155 to 4.764 for *Bacteria* and *Archaea*, respectively. The Gini inequality index ranged from 0.927 to 0.992 and from 0.959 to 0.993 for *Bacteria* and *Archaea*, respectively. The high Gini values obtained for both prokaryotic communities evidenced that they were characterized by low evenness, and in turn by the presence of few dominant OTUs.

**Table 2:** Alpha-diversity indices of the KKT bacterial and archaeal amplicon libraries

	*Bacteria*				*Archaea*		
Sample	Number of OTUs	Shannon Index	Gini Index		Number of OTUs	Shannon Index	Gini Index
KKT9	1379	4.718	0.965		517	4.452	0.979
KKT10	1176	4.505	0.972		448	4.056	0.983
KKT11	1044	4.132	0.977		422	3.832	0.986
KKT12	946	3.958	0.982		243	3.155	0.993
KKT13	890	3.941	0.983		252	3.247	0.993
KKT14	1049	3.984	0.977		339	3.335	0.991
KKT15	979	3.925	0.981		415	3.782	0.985
KKT16	716	2.686	0.992		396	3.881	0.986
KKT17	1944	4.861	0.944		514	4.156	0.978
KKT18	1816	5.455	0.933		1031	4.689	0.959
KKT19	1697	5.258	0.951		674	4.155	0.981
KKT20	1358	4.895	0.966		975	4.335	0.967
KKT21	1985	5.449	0.936		663	4.173	0.980
KKT22	2121	5.722	0.928		997	4.308	0.966
KKT23	1794	5.818	0.927		985	4.508	0.963
KKT24	2161	5.52	0.927		995	4.646	0.964
KKT25	2163	5.308	0.930		913	4.764	0.961
KKT26	1395	5.206	0.962		675	3.920	0.979

The depth and the hydrostatic pressure are key interrelated parameters, which are intrinsically fundamental in deep oceanic environments. To understand if differences in hydrostatic pressure exerted significant effects on KKT α-diversity, the GLMs were run to analyze whether the variation of indices according to the depth was significant. The analysis evidenced that for both bacterial and archaeal communities the α-diversity indices did not show significant variation (p > 0.05) in relation to this key-parameter.

**Fig. 7:**
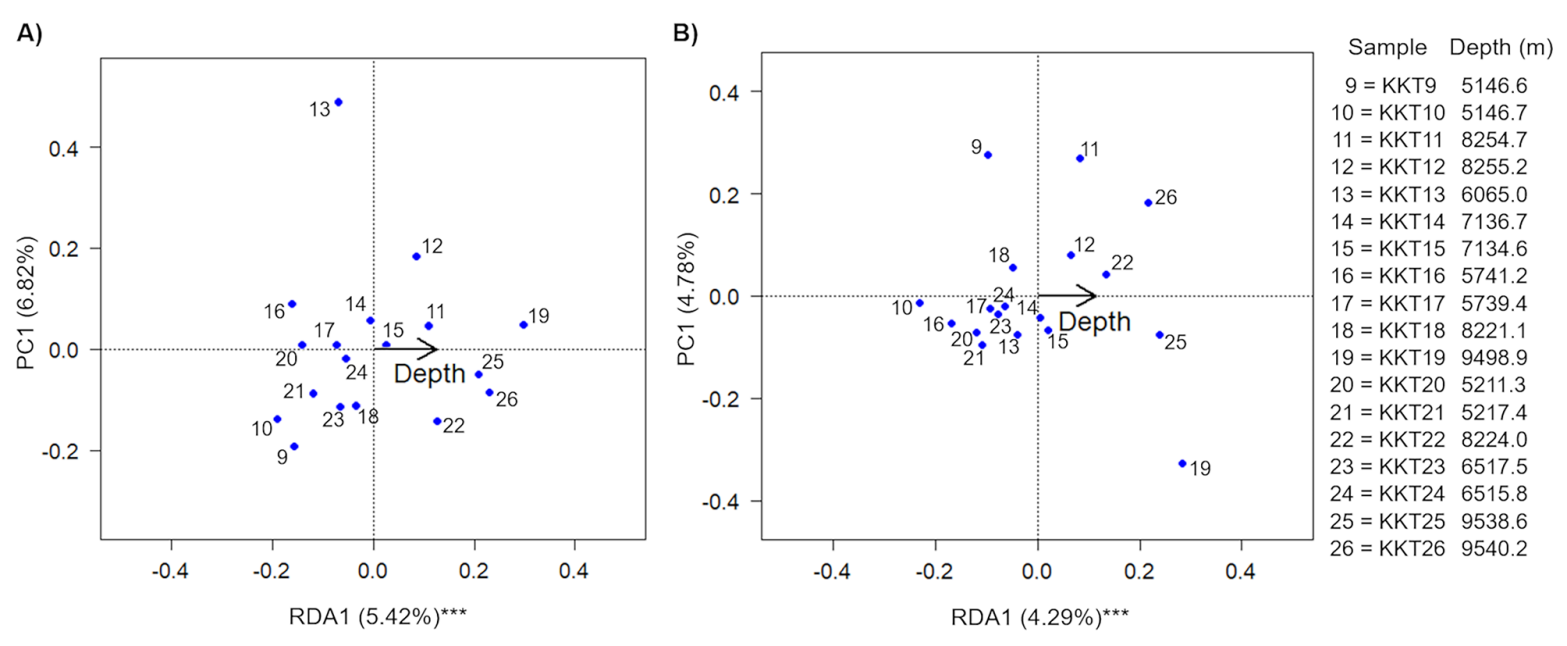
Redundancy analysis (RDA) ordination biplot showing the relationship between the KKT samples (based on bacterial (A) and archaeal (B) OTU profiles) and the depth

As expected, the RDA analysis revealed that the hydrostatic pressure clearly affected the whole structure of the prokaryotic communities. The KKT abyssal-hadal prokaryotic communities significantly differed (*Bacteria*: z = 3.373, P = 0.001, adjusted-R^2^ = 0.122; *Archaea*: z = 2.693, P = 0.001, adjusted-R^2^ = 0.091) according to the depth, which explained the 5.42% and 4.29% of data variability for the bacterial and archaeal community, respectively (Fig. 7).

Although no data are available for the considered ecosystem, it is possible to hypothesize that other variables in addition to the depth, such as nutrients, can explain the structuring of the KKT abyssal-hadal prokaryotic communities. Instead, as generally known, since the trench abyssal-hadal environments are quite stables, with temperature, light, and salinity that are always constant, it is reasonable to think that these environmental parameters do not represent key drivers for the community structure.

For *Bacteria*, the Cluster Analysis (based on Hellinger distance) identified five groups (Additional file1: Fig. S2). Based on their bacterial communities, unexpectedly, samples KKT25 (9538.6 m) and KKT26 (9540.2 m) grouped together with the samples KKT9 and KKT10, which were collected at the less deep layers (5146.6-5146.7 m). The samples collected in-between 8224 and 8254.7 m depth (KKT11, KKT12, and KKT22) clustered with those taken at 7134.6-7136.7 m (KKT14 and KKT15). Instead, KKT18 (8221.1 m) grouped with less deep samples (KKT16, KKT17, KKT20, KKT21, KKT23, and KKT24) that were collected at depths 5211.3-6517.5 m. Compared to these clusters, KKT13 (6065 m) and KKT19 (9498.9 m) showed the most divergent bacterial communities.

For *Archaea*, three main groups were identified through the Cluster Analysis (Additional file1: Fig. S3). Overall, KKT19 showed the most divergent archaeal community. A cluster included samples collected at the deepest layers (8224-9540.2 m), which grouped unexpectedly together with KKT21 (taken at 5217.4 m). Finally, a larger cluster included the samples collected at depths in-between 5146.6 and 8221.1 m.

### Bacterial and archaeal ecological function prediction

**Fig. 8:**
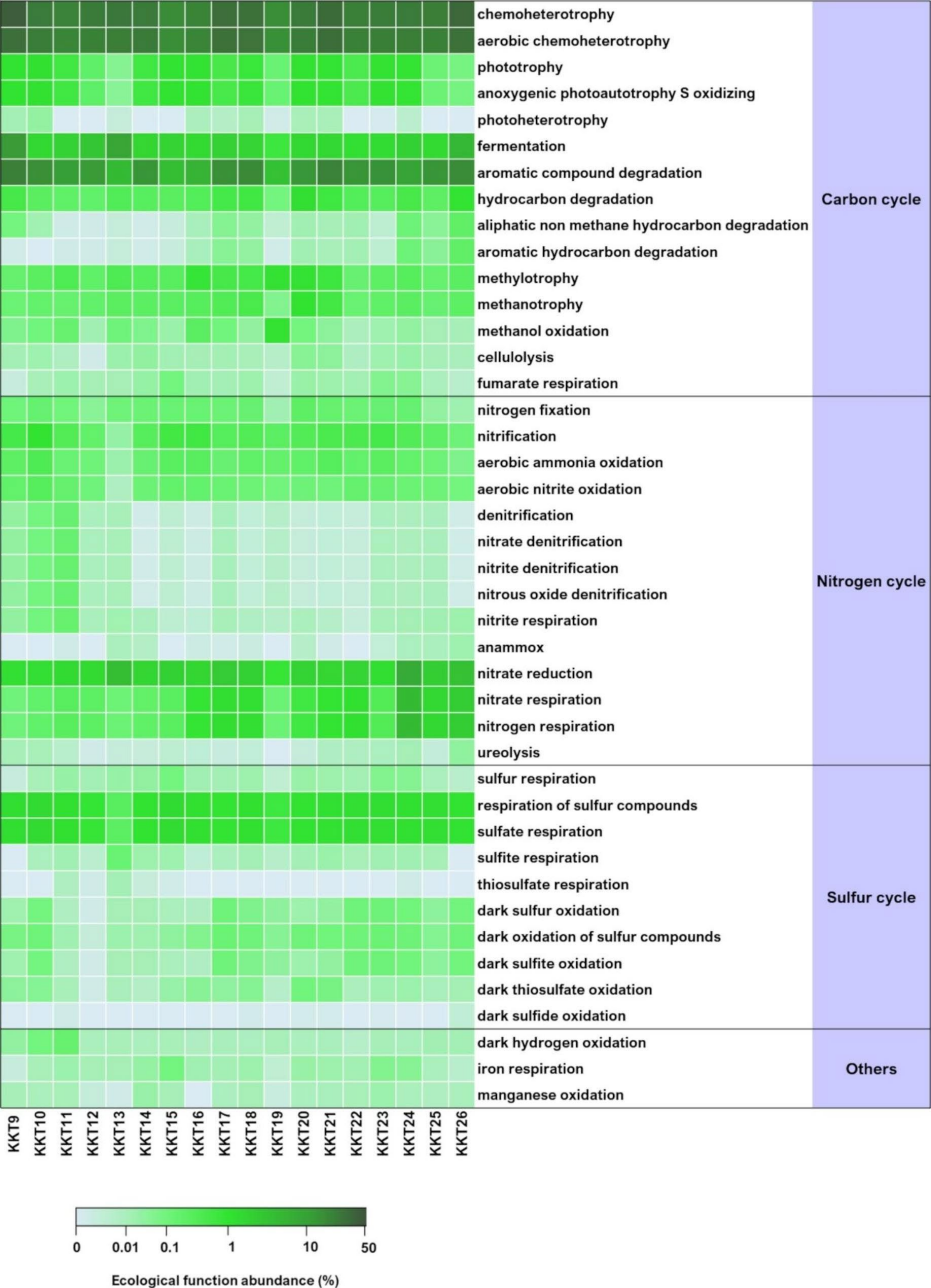
Ecological functions predicted for the KKT abyssal-hadal bacterial communities

**Fig. 9:**
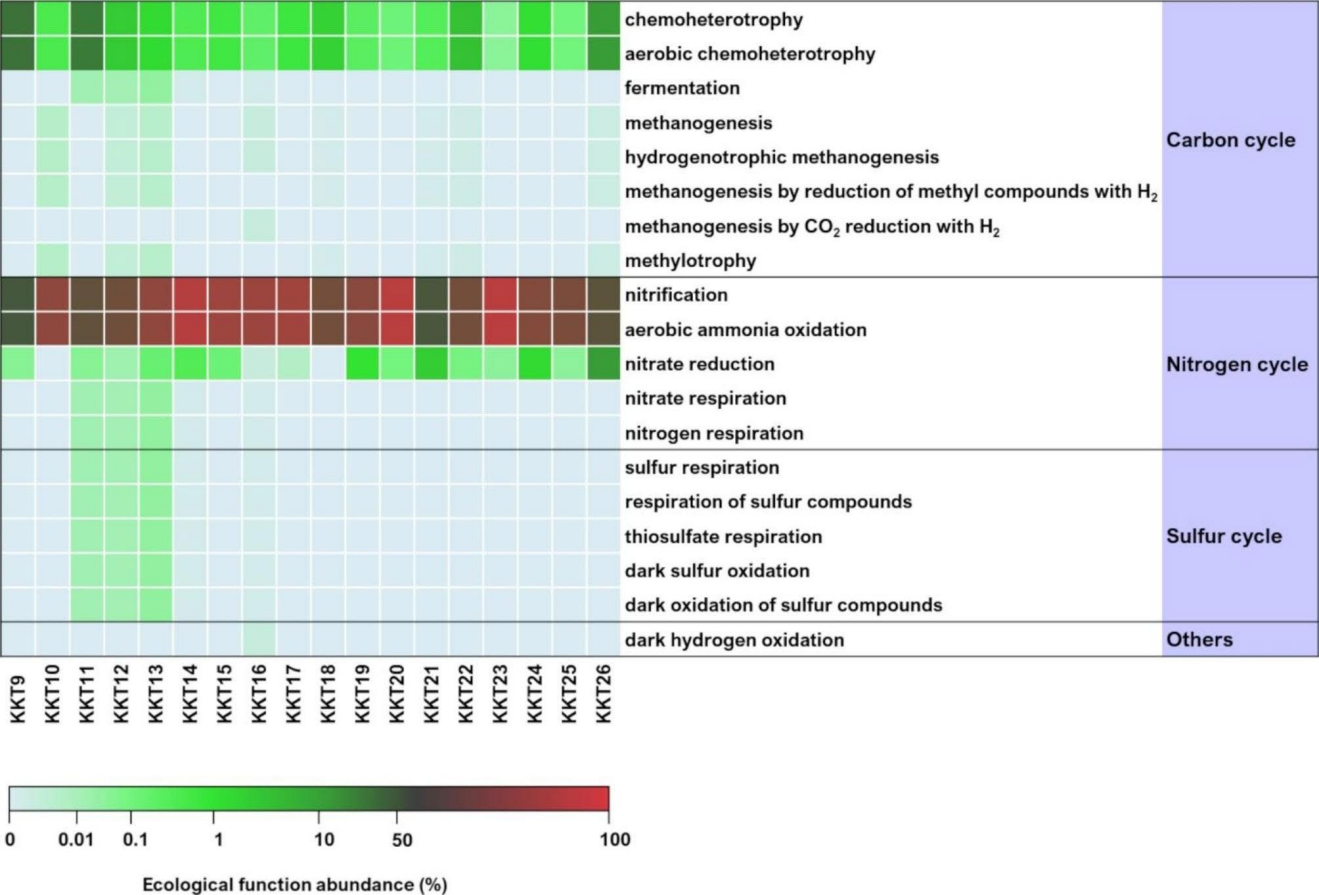
Ecological functions predicted for the KKT abyssal-hadal archaeal communities

The functional diversity analysis (FAPROTAX) showed that for both *Bacteria* and *Archaea* the predicted ecological functions were mainly associated with the carbon, nitrogen, and sulfur cycles (Figs. 8 and 9).

Chemoheterotrophy was the dominant metabolic strategy revealed in the bacterial communities and associated with 219 out of the 453 identified genera, including the aerobic chemoheterotrophic *Acinetobacter*, *Colwellia*, *Shewanella*, *Pseudomonas*, *Candidatus Pelagibacter*, and *Pseudoalteromonas*, which were among the major taxa found in KKT samples (Fig. 8; Additional file 2: Table S3; Fig. 2).

A rather high fraction, accounting for 5.4–27.3% of the whole bacterial communities, was characterized by aromatic compound degradative ability, related principally to *Acinetobacter* and to a lesser extent to *Gordonia*, *Nocardioides*, *Rhodococcus*, and *Sporobacter* (Fig. 8; Additional file 2: Table S3).

Among nitrogen cycle-related functions, nitrate reduction, nitrate respiration, and nitrogen respiration were the most represented predicted functions (in particular in KKT24, collected at 6515.8 m), largely associated with *Shewanella*; *Achromobacter*, *Aquabacterium*, *Azospira*, *Desulfuromusa*, *Paracoccus*, and *Stenotrophomonas* were involved to a lesser degree (Fig. 8; Additional file 2: Table S3).

As for the sulfur cycle, respiration of sulfur compounds and in particular sulfate respiration were the most predicted metabolisms, involving just minor genera such as *Desulfatiglans*, *Desulfatitalea*, *Desulfobacula*, and *Desulfobulbus* (Fig. 8; Additional file 2: Table S3).

Regarding the archaeal communities, the aerobic ammonia oxidation (the first step of the nitrification process) was the most represented ecological function, principally related to *Nitrosopumilus* and to a lesser extent to *Nitrososphaera* members (Fig. 9; Additional file 2: Table S4).

Also nitrate reduction (step of the denitrification process) was among the most predicted functions related to the nitrogen cycle, involving *Halorubrum*, *Natronomonas*, and *Thermodiscus* (Fig. 9; Additional file 2: Table S4).

A rather high fraction of the archaeal communities (in particular in KKT9 and KKT11 samples) was characterized by a chemoheterotrophyc metabolism, relating to *Halococcus*, *Methanomassiliicoccus*, *Natronomonas*, and *Thermodiscus* members (Fig. 9; Additional file 2: Table S4).

Metabolic functions related to the sulfur cycle were less represented and uniquely associated with the genus *Thermodiscus* (Fig. 9; Additional file 2: Table S4).

### Bacterial and archaeal OTU co-occurrence network

**Fig. 10:**
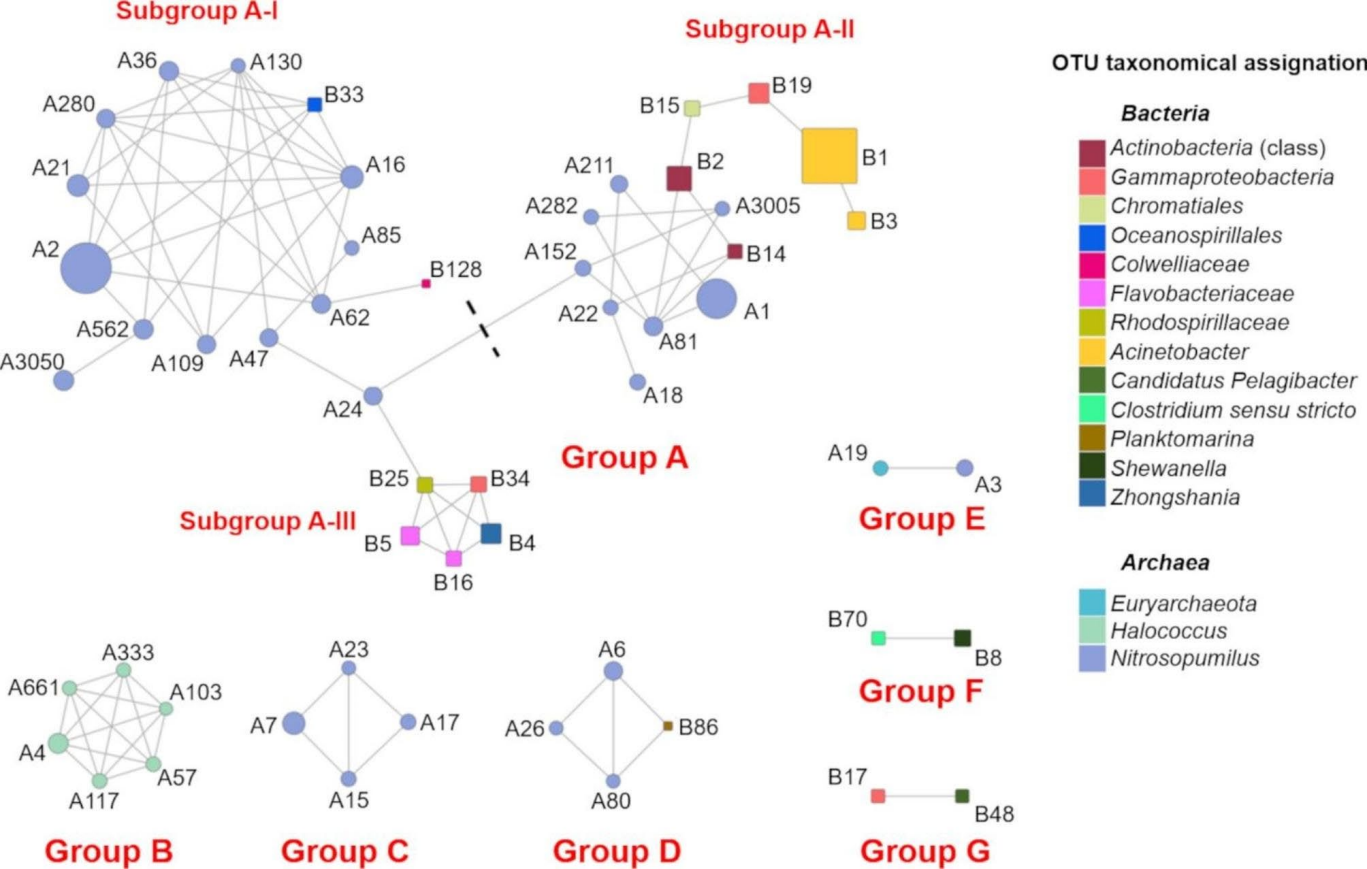
Co-occurrence network between bacterial and archaeal OTUs. Nodes represent the archaeal (circles) and bacterial (squares) OTUs, and edges (grey lines) represent the statistically significant positive correlations between each OTU pair; negative correlations are not shown. The node size is proportional to the mean relative abundance of OTUs in the dataset. The nodes are colored according to the taxonomic assignation (confidence ≥ 0.50)

The co-occurrence analysis was carried out to understand the interactions among the major bacteria and archaeal OTUs (Ra ≥ 1% in at least one sample) found in the benthic boundary layer of the KKT abyssal-hadal zone. The co-occurrence network (Fig. 10) showed seven groups (A-G) consisting of 2–34 OTUs (54 OTUs in total) and 1–60 edges.

There is a main group (group A) subdivided in 3 sub-groups linked by a connection node constituted by *Nitrosopumilus* OTU A24. This group included the principal (most abundant and ubiquitous in the KKT abyssal-hadal communities) archaeal and bacterial OTUs: *Nitrosopumilus* OTU A2 and OTU A1, (in subgroup A-I and A-II, respectively), *Acinetobacter* OTU B1 (in A-II). Except for two bacterial OTUs, belonging to *Colwelliaceae* (B128) and *Oceanospirillales* (B33), the main subgroup (A-I) showed a strong predominance of *Nitrosopumilus* OTUs. This subgroup was the highest connected module, with *Nitrosopumilus* OTU A2 and OTU A16 as the most interconnected nodes. Subgroup A-II consisted of various *Nitrosopumilus* OTUs connected with two bacterial OTUs. Among them, OTU B2 (class *Actinobacteria*) represented a node linking a peripheral core of bacterial OTUs. The subgroup A-III was the sole consisting of all bacterial OTUs. The groups B, C and E included only archaeal OTUs; among them, the most interconnected was Group B constituted by six *Halococcus* OTUs. Group D included three *Nitrosopumilus* and one *Planktomarina* OTUs. The groups F and G presented only two bacterial OTUs and connected respectively members of *Clostridium* with *Shewanella*, and *Gammaprotoeobacteria* with Candidatus *Pelagibacter*.

As expected (based on the main ecological functions predicted through FAPROTAX analysis), overall, the groups comprised ammonia-oxidizing archaea (*Nitrosopumilus*) and chemoheterotrophic bacteria and archaea (*Acinetobacter*, having also aromatic compound degradation abilities; *Shewanella*, involved also in nitrate reduction, nitrate and nitrogen respiration; *Candidatus Pelagibacter*; *Oceanospirillales* and *Colwelliaceae* members; *Halococcus*) (Additional file 2: Table S3 and Table S4).

It is not possible to find a strict relation between the various groups of the network and depth, being almost all OTUs detected at all depths. However, it is worth noting that, within the various groups/subgroups, most of the OTUs were recorded as major or even highly abundant at certain depths (Additional file 2: Table S5). For example, OTU A2 was always present with Ra > 4% and recorded with the highest abundances particularly in the range 5741-7135 m. All the A-II OTUs showed Ra ≥ 1% at the maximum depth (~ 9540 m), and most of them showed highest abundances in the hadal zone. Group C OTUs showed their highest abundances mainly in the KKT abyssal zone; whereas those of group D were major only in KKT19 (~ 9499 m). Group E was represented only by the archaeal OTUs A19 e A3, which showed their highest abundances in KKT22 (8224 m). The bacterial OTUs B8 and B70 in Group F showed highest abundances in KKT24 and KKT26 (~ 6516 m and ~ 9540 m, respectively). Group G gathers the bacterial OTUs B17 and B48, which were major just in one sample (KKT19, ~ 9499 m) with quite similar abundances.

## Conclusion

Summarizing, the present work allowed portraying the bacterial and archaeal communities of the KKT abyssal-hadal zone, which were never investigated before. Although the availability of environmental parameters to relate with the studied prokaryotic diversity would have provided a more informative ecological overview, the work represents an important contribution to the scientific community, especially if taking into account that no further explorations of the area are likely to be organized probably for many years to come. The survey evidenced that this extreme environment harbored communities that differed from those of other marine trenches. The highly uneven KKT bacterial and archaeal communities were mainly represented by heterotrophic and chemolithotrophic genera, respectively, which did not show a distinctive zonation but were detected at all depths. In particular, *Acinetobacter*, *Zhongshania*, and *Colwellia* (potential hydrocarbon degraders) were the main bacterial genera, and *Nitrosopumilus* (ammonia oxidizer) *was* the dominant archaeal representative of the KKT deepest areas.

## Electronic supplementary material

Below is the link to the electronic supplementary material.

Additional file 1: Fig. S1. Rarefaction curves of KKT abyssal-hadal samples for *Bacteria* (A) and *Archaea* (B) libraries; Fig. S2. Cluster Analysis (*Bacteria* dataset) of the KKT abyssal-hadal samples based on Hellinger distance; Fig. S3. Cluster Analysis (*Archaea* dataset) of the KKT abyssal-hadal samples based on Hellinger distance.

Additional file 2: Table S1. Bacterial genera found in the abyssal-hadal zones of the KKT and other trenches; Table S2. Archaeal genera found in the abyssal-hadal zones of the KKT and other trenches; Table S3. Bacterial ecological functions predicted through FAPROTAX analysis; Table S4. Archaeal ecological functions predicted through FAPROTAX analysis; Table S5. Abundance of co-occurrent bacterial and archaeal OTUs (network analysis) across the KKT abyssal and hadal samples.

## Data Availability

The datasets generated during and/or analyzed during the current study are available in the Sequence Read Archive (SRA; https://www.ncbi.nlm.nih.gov/sra) repository (SRA accession numbers: PRJNA799316 and PRJNA799327).

## List of Abbreviations

KKT: Kuril–Kamchatka Trench
OTU: Operational Taxonomic Unit
RDA: Redundancy Analysis
GLM: Generalized Linear Model
FDR: False Discovery Rate
Ra: Relative abundance
