# Supplementary material for: Insights into the prokaryotic communities of the abyssal-hadal benthic-boundary layer of the Kuril Kamchatka Trench
Source: Environ Microbiome. 2023 Aug 2;18:67. doi: 10.1186/s40793-023-00522-9 (PMC10398949; doi:10.1186/s40793-023-00522-9)
Supplement: Supplementary file 1 — Additional file 1: Fig. S1. Rarefaction curves of KKT abyssal-hadal samples for Bacteria (A) and Archaea (B) libraries; Fig. S2. Cluster Analysis (Bacteria dataset) of the KKT abyssal-hadal samples based on Hellinger distance; Fig. S3. Cluster Analysis (Archaea dataset) of the KKT abyssal-hadal samples based on Hellinger distance. [file 40793_2023_522_MOESM1_ESM.docx]

**Additional file 1**

Insights into the Prokaryotic Communities of the Abyssal-Hadal benthic-boundary layer of the Kuril Kamchatka Trench

Susanna Gorrasi^1*^, Andrea Franzetti^2^, Angelika Brandt^3,4^, Ulrike Minzlaff^4^, Marcella Pasqualetti^1,5^, and Massimiliano Fenice^1,6*^

^1^Laboratory of Microbiology, Department of Ecological and Biological Sciences, University of Tuscia, Viterbo 01100, Italy

^2^Laboratory of Microbiology, Department of Earth and Environmental Sciences, University of Milano-Bicocca, Milano 20126, Italy

^3^Senckenberg Research Institute and Natural History Museum, Frankfurt am Main 60325, Germany

^4^Institute of Ecology, Diversity and Evolution, Goethe University, Frankfurt am Main 60438, Germany

^5^Laboratory of Ecology of Marine Fungi - CoNISMa, Department of Ecological and Biological Sciences, University of Tuscia, Viterbo 01100, Italy

^6^Laboratory of Applied Marine Microbiology - CoNISMa, Department of Ecological and Biological Sciences, University of Tuscia, Viterbo 01100, Italy

***Correspondence:**Massimiliano Fenice, fenice@unitus.it; Susanna Gorrasi, gorrasi@unitus.it


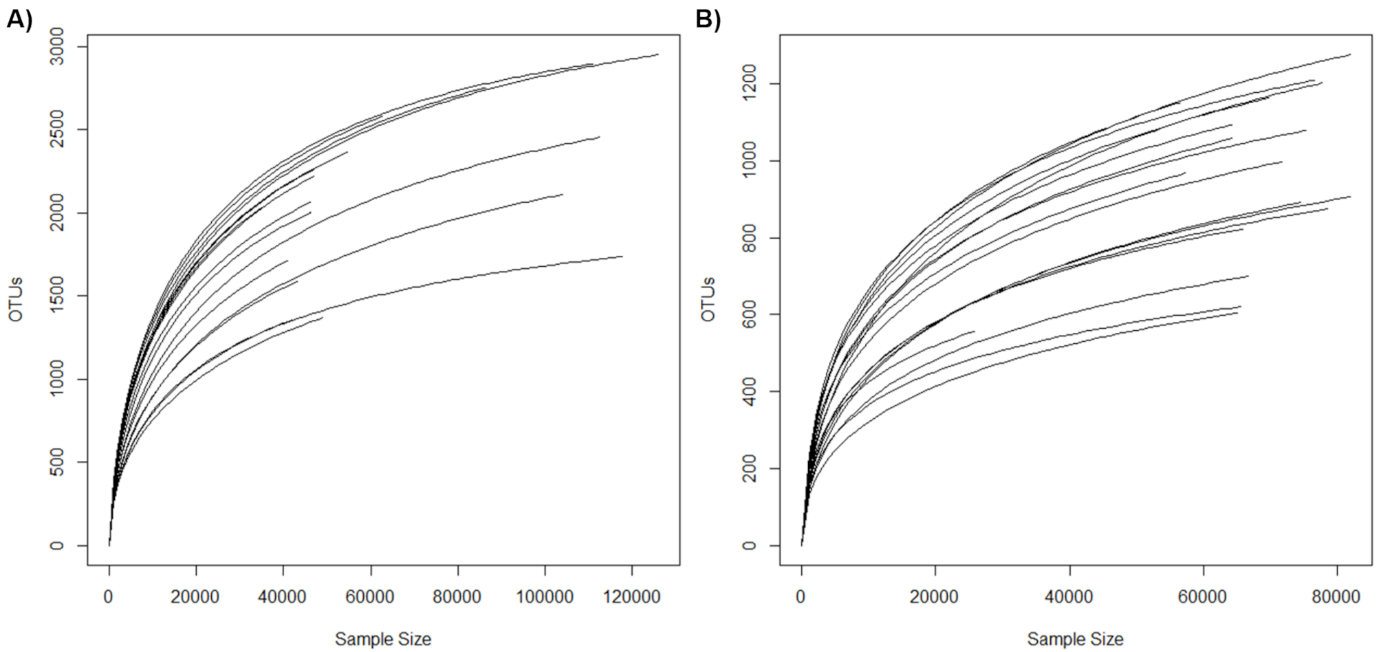


**Figure S1.** Rarefaction curves of KKT abysso-hadal samples for *Bacteria* (A) and *Archaea* (B) libraries.


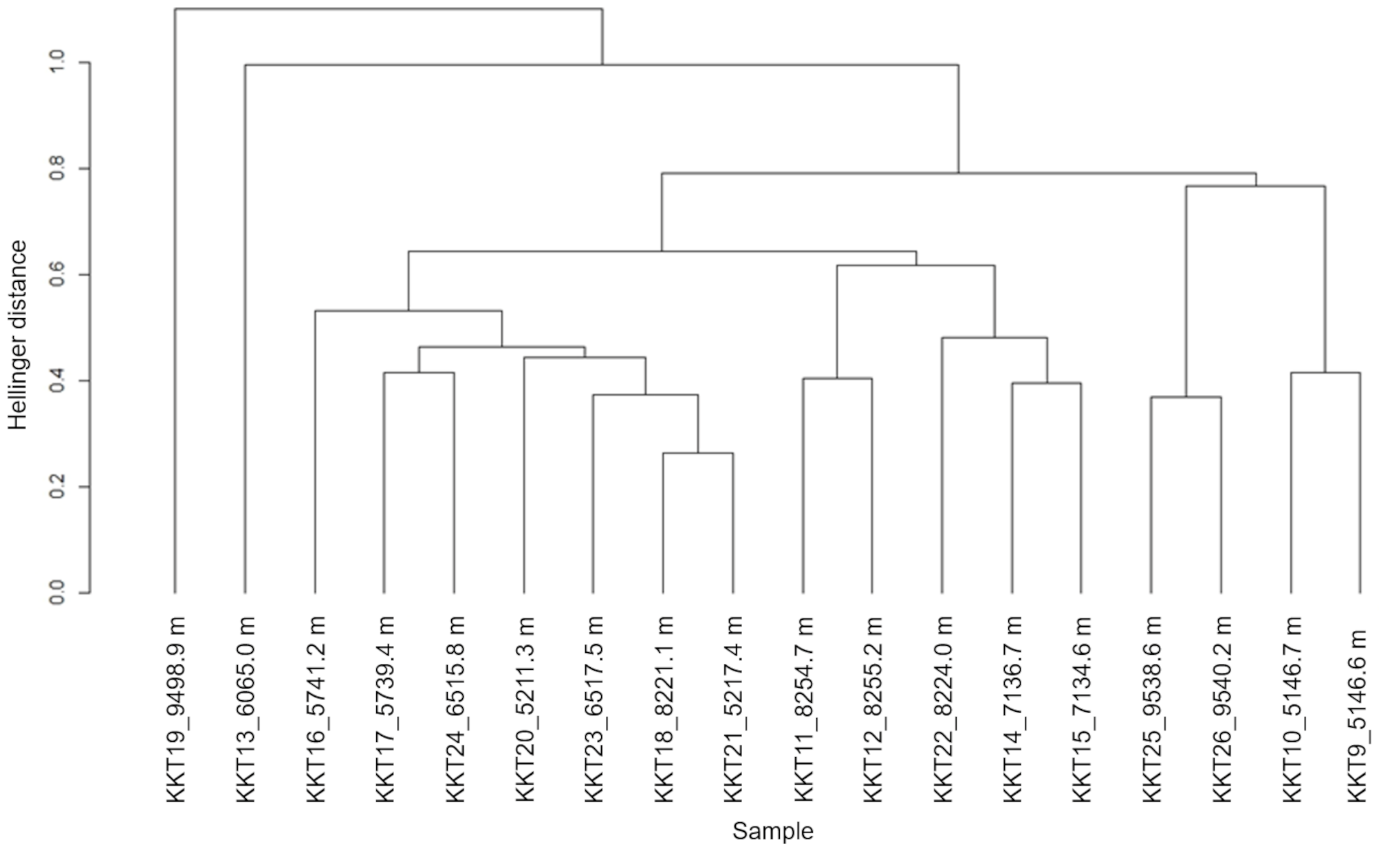


**Figure S2.** Cluster Analysis (*Bacteria* dataset) of the KKT abysso-hadal samples based on Hellinger distance.


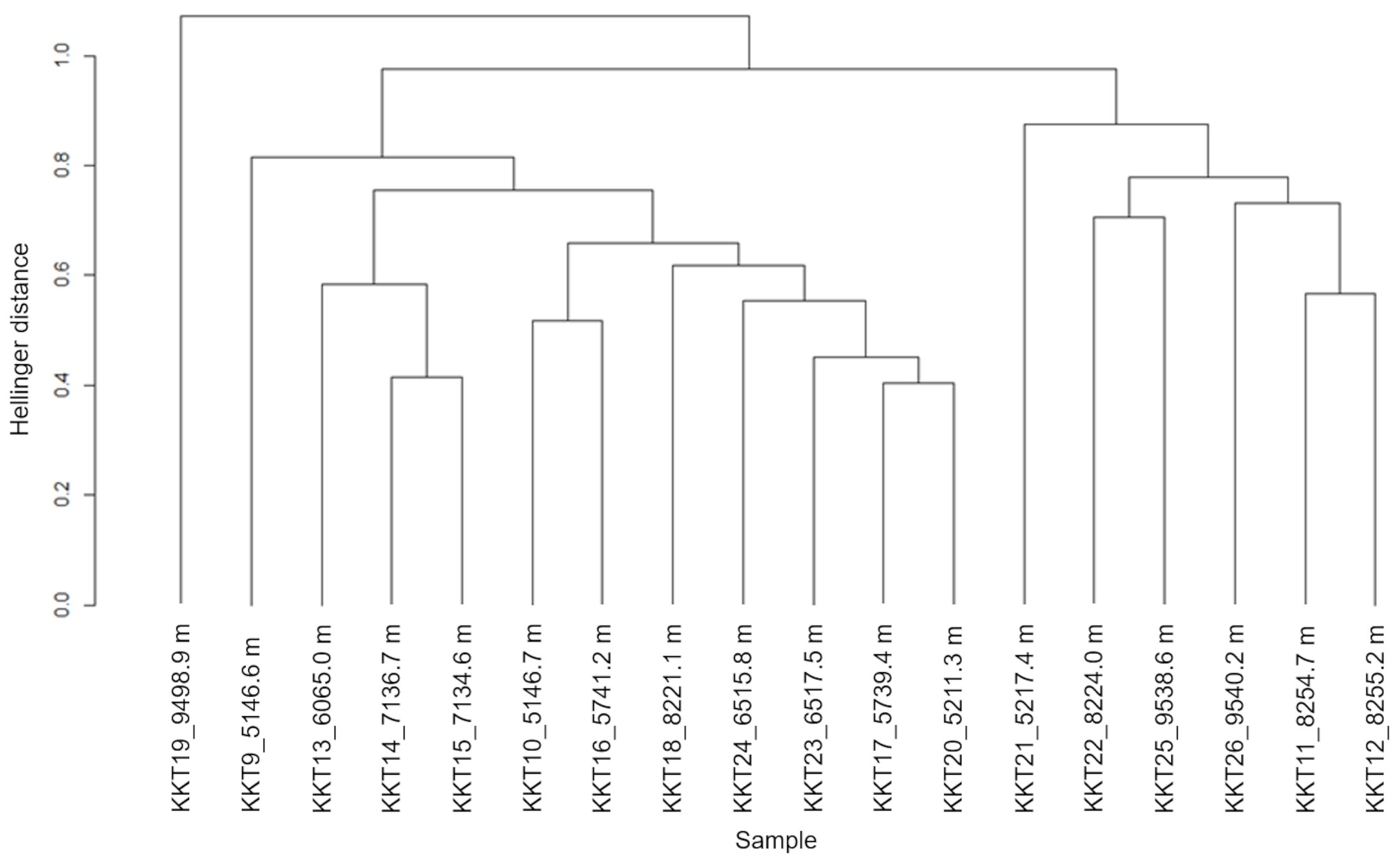


**Figure S3.** Cluster Analysis (*Archaea* dataset) of the KKT abysso-hadal samples based on Hellinger distance.
